# Articles

**Published:** 1841

**Authors:** A. Hill


*Norwalk, Ct. April 23rd,* 1841.

DR. SOLYMAN BROWN,

DEAR SIR—I herewith transmit to you, an account of that remarkable case of Salivary Calculi to which my attention was solicited some time since; the specimens of which I exhibited to you in N. York not long ago. And I very much regret, that a want of time should prevent that thorough investigation of the subject that so remarkable a case seems to require.

Many valuable and elaborate treatisies have been written, and much ingenuity has been exhausted, on the subject of Urinary Calculi. Stores of learning, and much treasured thought have been spent, in trying to investigate and understand the nature of that peculiar constitutional diathesis which predisposes to this painful disease. But as yet I have seen comparatively little on the kindred subject of salivary calculi—I say kindred subject, for I can scarcely resist the conviction that the same diathesis predisposes to both these complaints, and that a peculiar irritability, similar in its nature in both organs, invites disease, either to the one part or the other. I am led to this conclusion, not only from the analogy that exists between the two organs, that are the seats of disease, but from the similarity of diseased action in both—both secreting the phosphate of lime, and the earthy salts, in combination with different acids in a remarkable manner,—and the diseased action of either, or both, accompanied with a loose strumous habit.* Indeed so strikingly manifest has this been in many cases, that I have been led to conclude, that in all similar instances there is a predominant glandular irritation throughout the system; and that the primary cause of this disease, may be found in that which produces a vitiated state of the secretions.—I cannot stop here to enquire into all the causes, that may have induced this derangement in the secretory organs. It is sufficient at present to observe, that in very many instances, it appears as the sequel of a course of mercurial medicines, that have been administered, as remedial agents, in cases of disease. And now, if there be any propriety or correctness in the view which we have here taken of this complaint, brief as it may be, it will suggest to us a rational system of treatment; and strange as it may seem, such a system of treatment is yet a desideratum in medical literature. And if a treatment of a local character merely, was all that demanded the attention of the dental practitioner, and no knowledge of the human system, or the nume-

* It is conceded by our most eminent writers, on both urinary and salivary calculi, and the opinion is confirmed by common observation, that bilious and scorbutic habits are by far the most subject to affections of this kind. The characteristics, however, of these earthy concretions, vary in different temperaments, so that a person at all exercised in this sort of inquiry, can, by their inspection, tell the habit of the individual from which they are taken.—EDS.

rous morbid changes to which it is liable, was necessary, then indeed, would there be a more plausible excuse for the palpable ignorance of so many who assume the important duties of the surgeon dentist. But as the treatment of these diseases very properly comes under his care, and within the appropriate sphere of his practice, and the treatment not merely local, but constitutional, a tolerably correct knowledge of anatomy, physiology, pathology and therapeutics, seems indispensable; for without it he will be constantly exposed to the charge of quackery, and downright charlatanism—nor will the opprobrium be misapplied.

But to the case: — Sometime last fall, a lady called on me, expressing a desire that I should examine her mouth, and stated at the same time that "something was growing there exceedingly troublesome." On examination I found that a solid mass of calcarious matter, extended from the sub-linguial glands back to the angle of the jaw; and in one continuous mass from the articulation of the jaw to the superior bicuspids, on the right side, and also on the lower jaw, extending from the root of the tongue to the tops of the teeth, in width nearly one inch. I forthwith removed it from the mouth, and found it smoothe on its upper side, but presenting a rough and sponge-like appearance on its lower surface, and of a greenish colour. The gums were very much inflamed, loose, and flabby. A large abscess had previously formed on the outside of the face, corresponding with the mass on the inside, and much pain, and iritation, was the consequence. The patient was about forty years of age, and of a decidedly strumous habit. She stated to me, that it was about three years since it began to accumulate in this way; and that during that period, she had removed several pieces from her mouth. I have estimated the weight of that portion which I removed from the mouth, at seventy-eight grains.

By local applications, and constitutional remedies, I have succeeded in subduing that strong tendency of the system to this disease.

I remain, very respectfully, yours, &c. A. HILL.

